# Bio-based polyurethane nanocomposite thin coatings from two comparable POSS with eight same vertex groups for controlled release urea

**DOI:** 10.1038/s41598-021-89254-9

**Published:** 2021-05-10

**Authors:** Lixia Li, Meng Wang, Xiandong Wu, Wenping Yi, Qiang Xiao

**Affiliations:** 1 Institute of Plant Nutrition and Resource, Beijing Academy of Agriculture and Forestry Sciences, Beijing, 100097 China; 2 Research Center of Beijing Municipal Slow and Controlled Release Fertilizers Engineering Technology, Beijing, 100097 China; 3 School of Nuclear Science and Technology, University of South China, Hengyang, 421001 China

**Keywords:** Biomaterials, Nanoscale materials

## Abstract

Nanocomposite modification has attracted much attention in improving properties of bio-based polymer coating material for coated fertilizer. Herein two comparable polyhedral oligomeric silsesquioxanes (POSS), with eight poly(ethylene glycol) (PEG) and octaphenyl groups attached to the cage, respectively, were successfully incorporated into thin castor oil-based polyurethane coatings via in-situ polymerization on the urea surface. The nanostructure coatings are environmentally friendly, easy to prepare, and property-tunable. The results show that the vertex group of POSS had a pronounced influence on dispersion level and interaction between polyurethane and POSS that well-tuned the release pattern and period of coated urea, even at the coating rate as low as of 2 wt%. The liquid POSS with long and flexible PEG groups had better compatibility and dispersibility in polyurethane matrix than the solid POSS with rigid octaphenyl groups, as evidenced by SEM/EDS. The unique properties were resulted from the different extents of physical crosslinkings. This modification of bio-based polyurethane coating with POSS provided an alternative method of regulating and controlling the properties of coated fertilizer.

## Introduction

Coated fertilizer is widely used in agriculture because it increases nutrient utilization efficiency and decreases pollution to air and groundwater^1–3^. Its nutrient release pattern demonstrates a strong dependence on the used coating material^4,5^. Polyurethane (PU) made by the reaction of polyol and isocyanate is a kind of block copolymer composed of soft chain segment and hard chain segment. By changing the composition and proportion of the two phases, its microstructure is adjusted and the specific property is improved. Therefore, currently polyurethane coatings are favored by the researchers and manufacturers of non-solvent in-situ reaction coated fertilizers^6–8^.

Bio-based polyurethane coatings from natural materials such as starch^9^, lignin^10^, cellulose^11^ and vegetable oil^12^, are considered to be green, sustainable and low-cost. Thus they have shown great potential as a substitute for conventional synthetic polymer coatings. Bortoletto-Santos et al*.*^13^ prepared castor oil-based polyurethane coated fertilizer. The nitrogen release period of fertilizer with the coating rate of about 4 wt% was 40 days. Zhang et al*.*^14–16^ designed a series of coated fertilizers by curing liquefaction products of starch, wheat straw and waste paper to the fertilizer surface, and the resulted coatings had the good biodegradability in soil. The effect of coating microstructure on fertilizer performance is particularly obvious especially for the ultrathin coating. Therefore, the researchers tried some methods to optimize the microstructure, such as blend and nanocomposite^17–19^. Reportedly Yang et al*.*^20,21^ modified the bio-based polyurethane coating material using epoxy or polyester resin to form interpenetrating network structure, which enhanced the slow-release properties of coated fertilizers. Zhao et al*.*^22^ filled nanobentonite to the soybean oil-based polyurethane coating. When the addition content of polyethylene glycol-intercalated bentonite was 5 wt%, the nutrient release period of coated urea was 74 d. Till now, when the coating rate of fertilizer was lower than 2.5 wt%, the nutrient release period was not longer than 30 d yet. A simple and efficient approach still brings a challenge in designing microstructures and tuning release properties of fertilizers with ultrathin bio-based polyurethane coatings.

The synthesis of nanomaterials gains growing attention^23,24^, which offers alternative choices for the microstructure optimization of coatings. POSS, a cagelike molecule containing RSiO_3/2_ units, is an environmentally friendly nanomaterial developed in recent years^25^. Not only does POSS combine the advantage of both inorganic and organic components, but it also has some new properties produced by synergistic effect^26^. POSS with eight PEG groups attached on the cage (POSS-PEG) was ever used to modify MDI-PTMG-based polyurethane. The POSS-PEG dispersed well on the molecular level and acted as a diluent to plasticize the molecular dynamics^27^. Huang et al*.*^28^ prepared an environmentally friendly polyurethane nanocomposite with POSS as a chain extender. Its surface hydrophobicity was significantly improved in comparison with that without POSS. What’s more, it was reported that the degradation controllability of polyurethane coating for controlled release of drug was improved by the incorporation of POSS^29,30^. To our best knowledge, the effect of POSS on the release behavior of fertilizer from polymer coated fertilizer is an unknown field. Since the preparation process and raw material type directly affect the combination mode of nanoparticle with polymer matrix and the topology of nanocomposite^31,32^, it is necessary to specially conduct the in-depth research on the solvent-free in-situ polymerization of bio-based polyurethane coating on the fertilizer surface.

**Figure 1:**
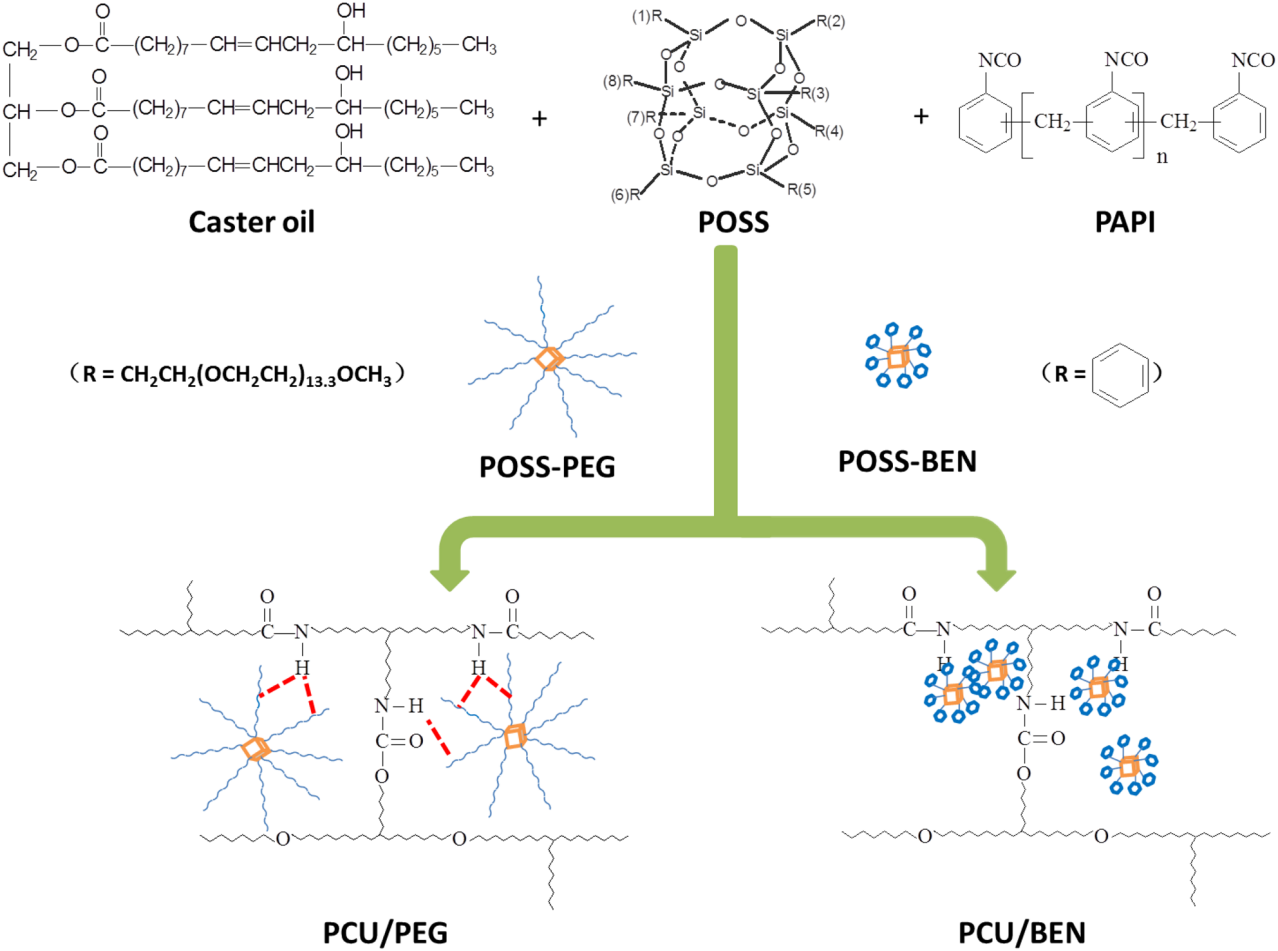
Formation illustration of nanocomposite coatings.

In the present study, the castor oil-based polyurethane nanocomposite coating containing the commercial liquid POSS-PEG was firstly designed. Wherein the PEG vertex groups possess the same repeated unit of –CH_2_CH_2_O– as polyether polyols, and the size of POSS-PEG is longer than that of silicon cage or castor oil. PEG vertex groups improve the compatibility by the hydrogen bonding interaction between the hydroxyl groups on castor oil and the ether oxygens on PEG. For comparative purpose, POSS with octaphenyl groups (POSS-BEN) was selected, where no chemical links bound the POSS molecules to polyurethane matrix. The formation of the nanocomposite coatings is illustrated in Fig. 1.

## Results and discussion

**Figure 2:**
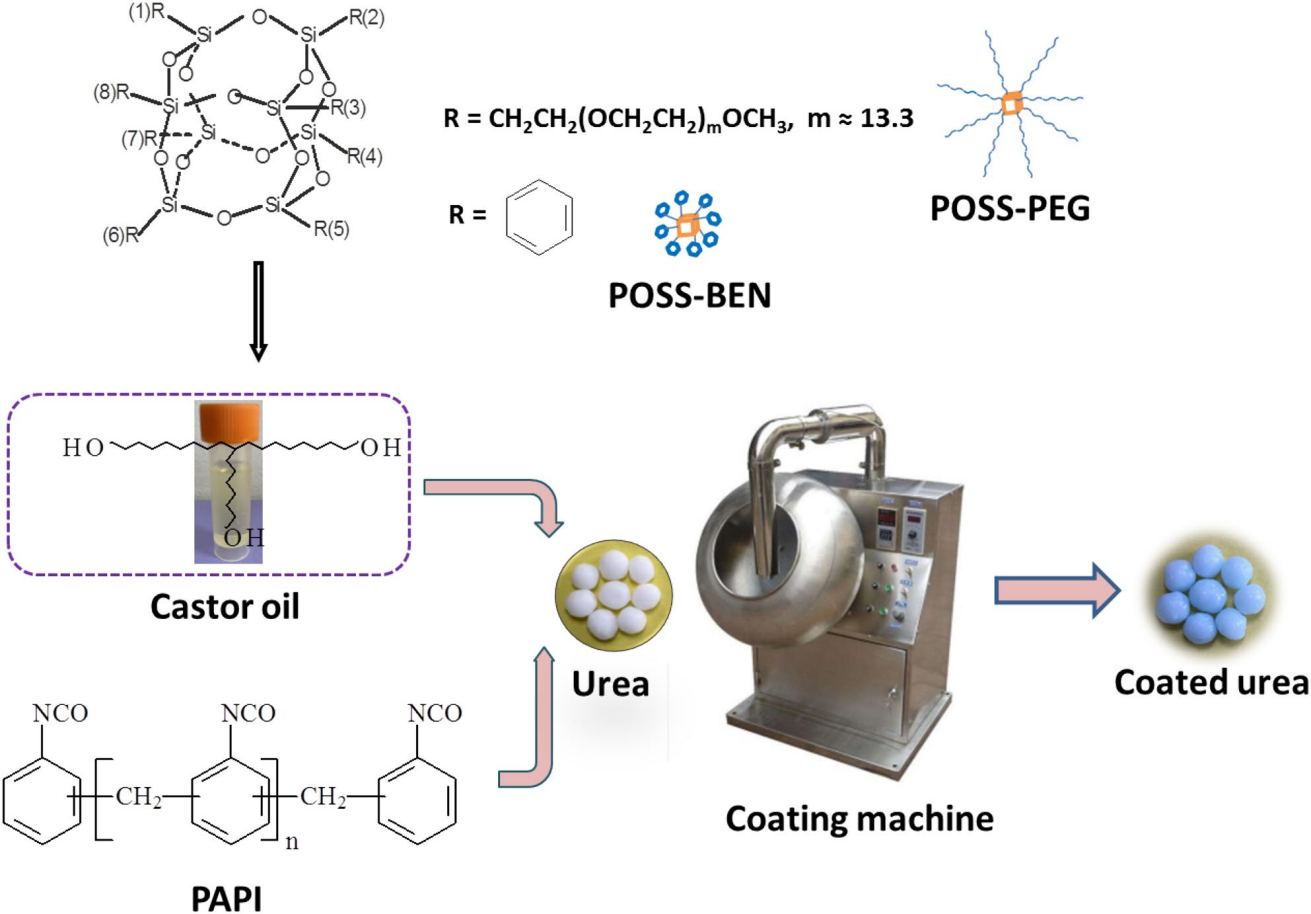
The preparation process of nanocomposite coated urea. (Photographs of urea, castor oil and coated urea were taken by us, and photograph of coating machine was provided by Beijing Health and Medical Equipment Co., Ltd. China).

In the study, two comparable polyurethane/POSS nanocomposite coatings were synthesized via in-situ polymerization on the urea surface. The preparation process of the nanocomposite coated urea is shown in Fig. 2. The resultant coated urea granules are designated as PCU/BEN*n* and PCU/PEG*n* (*n* refers to the weight percentage of POSS in the coating liquid). A control sample designated as PCU was prepared in a similar manner except that pure polyurethane was used to coat the urea granules. Water resistance and controlled release properties of the coatings were evaluated. The exact interaction mechanism of POSS and PU matrix was further investigated by a combination of thermal, mechanical and morphological analyses.

### FTIR of POSS

**Figure 3:**
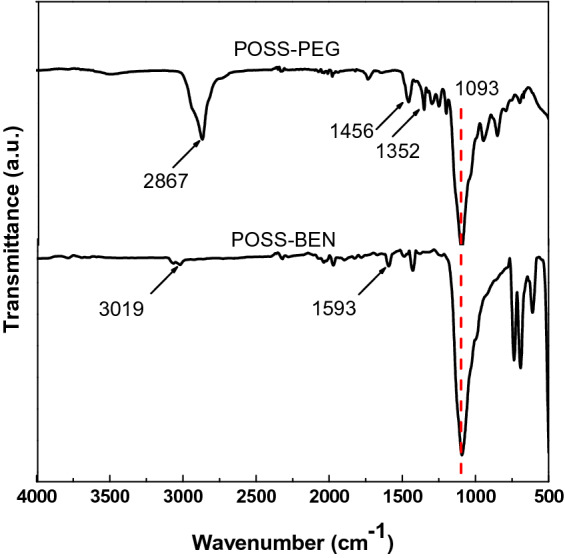
FTIR spectra of POSS with eight PEG groups (POSS-PEG) and POSS with octaphenyl groups (POSS-BEN).

Figure 3 shows the FTIR spectra of pure POSS-PEG and POSS-BEN. Each sample exhibited an absorption band at 1093 cm^−1^ corresponding to the Si–O–Si bond, which is the characteristic signal of POSS^33^. The characteristic absorption band was further assigned to stretching vibration of C–O bond of PEG chain^34^. The peak of 2867 cm^−1^ was associated with the CH_2_ and CH_3_ symmetric and antisymmetric stretching vibrations, and the peaks located at 1456 cm^−1^ and 1352 cm^−1^ were attributed to C–H bending vibrations, which indicated the existence of PEG chain. In the case of POSS-BEN, both 3019 cm^−1^ and 1593 cm^−1^ were the characteristic absorption peaks of benzene ring framework.

### TG/DTG of coatings

**Figure 4:**
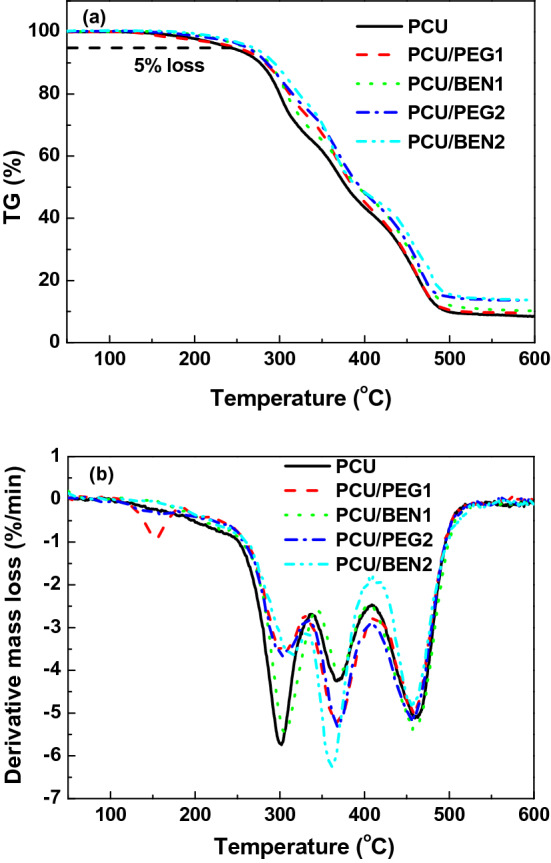
TG (**a**) and DTG (**b**) curves of coatings.

TG/DTG was performed to study the thermal properties of the coating materials. The curves are shown in Fig. 4 and Table 1 summarizes the corresponding data. It is noticed from Fig. 4a that the structural weigh loses of PCU coating began at around 242 °C, lower than that of nanocomposite coatings, implying the better thermal stability of nanocomposite coatings. As seen from Fig. 4b, the thermal decomposition processes of coating materials were divided into three stages at elevated temperatures, i.e. the degradation of hard segments (e.g. urethane bond) into isocyanate and polyol, the degradation of soft segments (e.g. polyol backbone) and the degradation of chars formed in the previous steps, which demonstrated that the introduction of POSS did not alter the degradation mechanism of polyurethane matrix. The two types of POSS acted as nanofillers similar to common fillers, but the interaction of PU with POSS was different. The hydrogen bond existed between N–H of hard segments and ether oxygen on POSS-PEG. Thus the long and flexible PEG segment played a role of physical crosslinking to twine with polyurethane, which limited the movement of polyurethane segment. The aromatic ring with high rigidity was looked on as rigid particle and slowed down the movement ability of polyurethane segment. Besides, after blending with the same POSS content, the residual weight of PCU/BEN increased a bit more than that of PCU/PEG, which was resulted from the higher content of silicon for POSS-BEN. The coatings are also analyzed by XRD and FTIR. The results are shown in Fig. S1-2. Considering the microstructure of nanocomposite depends on POSS content, here the maximum of POSS content is 2 wt% and thus the change is not detectable.

**Table 1:** Characteristic data obtained from TG/DTG curves of coatings.

Coating	T_5%_ (°C)^a^	T_1_ (°C)^b^	T_2_ (°C)^c^	T_3_ (°C)^d^	Residual weight (%)
PCU	242	301	368	459	8.8
PCU/PEG1	250	303	368	459	9.7
PCU/BEN1	261	306	370	459	10.5
PCU/PEG2	262	304	369	458	13.0
PCU/BEN2	268	315	363	456	14.1

^a^Temperature for 5% mass loss, ^b–d^Temperature for the mass loss rate.

### DMA of coatings

**Figure 5:**
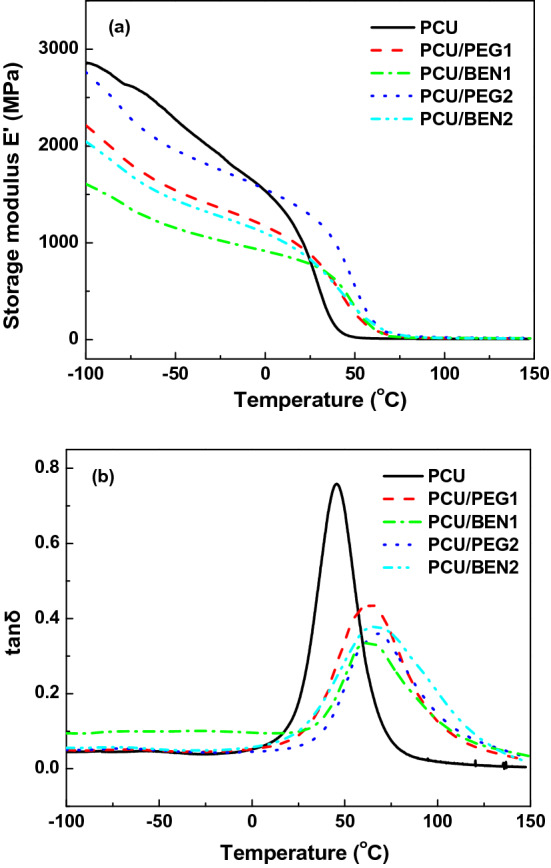
Storage modulus E′ (**a**) and tanδ (**b**) over temperature for coatings.

The DMA spectra were performed to explore the effect of POSS on storage modulus E′ and tanδ of the coating materials. Figure 5a shows that below room temperature the coating materials were in glassy state and the molecular chains inside the coatings were frozen. Above room temperature the storage modulus rapidly decreased, which meant that the materials entered the glass transition region and the molecular chains began to move. Notably, at or near room temperature the storage modulus of the nanocomposite coatings filled with POSS was higher than that of pure PCU, suggesting that POSS was favorable to increase the stiffness. A single peak was observed in each film shown in Fig. 5b and the peak value corresponded to their Tg. It can be explained that the microphase separation of PU was not evident, and the high miscibility of POSS in the matrix was attributed to the organic PEG side chains of POSS-PEG and aromatic rings of POSS-BEN as designed. Similar result was obtained by Gama et al.^35^. Besides, the shape and position of tanδ peaks were obviously affected by POSS. Compared with pure PCU, the peaks of composite coatings clearly revealed POSS-relevant broadening, which implied that the interaction of POSS with PU introduced the heterogeneity. The Tg of PCU was 42 °C while that of composite coatings was increased up to above 50 °C. The shift of Tg was due to the formation of more physical crosslinking points from hydrogen bond interaction, formation of POSS domain and so on^36^, which restricted the movement of PU chain and POSS cage.

### Morphology of coatings

**Figure 6:**
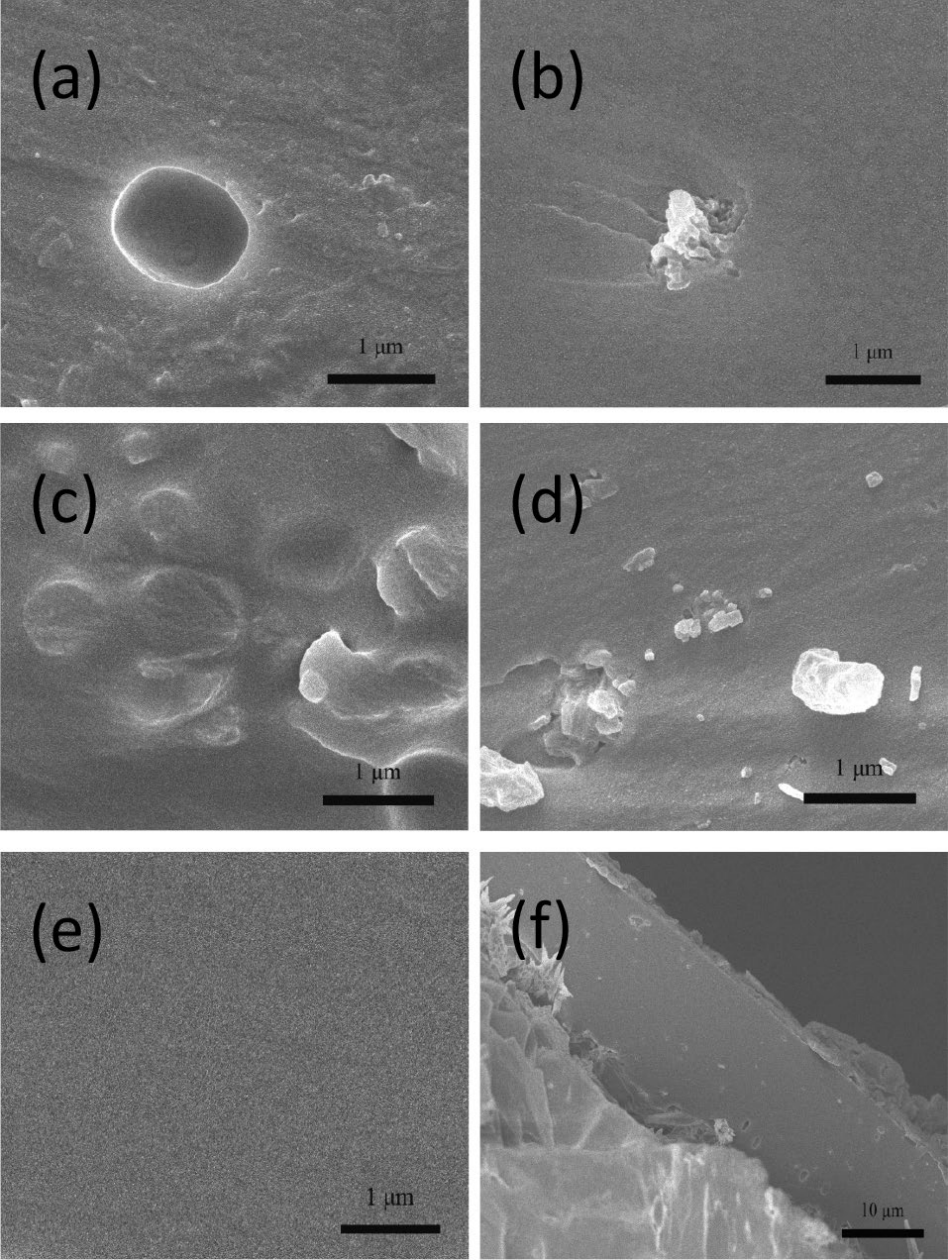
SEM images of cross-section morphologies of coatings. PU/1 wt% POSS-PEG nanocomposite (PCU/PEG1) (**a**), PU/1 wt% POSS-BEN nanocomposite (PCU/BEN1) (**b**), PU/2 wt% POSS-PEG nanocomposite (PCU/PEG2) (**c**), PU/2 wt% POSS-BEN nanocomposite PCU/BEN2 (**d**), pure polyurethane (PCU) (**e**) at × 20,000 magnification and (**f**) PCU/PEG1 at × 2000 magnification.

To follow the effect of POSS on PU matrix, the cross-section morphologies of the coatings were studied by SEM and are depicted in Fig. 6a–f. When the addition content was 1 wt%, the coatings were no longer as smooth as PCU, but regardless of POSS type a good dispersion was observed. In the case of PCU/PEG1, the fracture surface became rough, which exhibited a ductile fracture^37^. PEG vertex groups improved the compatibility between POSS-PEG and PU. POSS-BEN was surrounded by PU matrix to form a sea-island structure, which indicated a strong affinity^38^. It is worthy noted that after the POSS-BEN number increased with the loading increase, some particles were aggregated into a larger size than 1 μm. The detached particles and holes appeared as a result of a strong aggregation effect of POSS-BEN through physical interactions assigned to benzene ring being hydrophobic and inert to PU similar to the report by Cheng^39^. The coating part was involved in reducing the rates of both water diffusion into the core and urea diffusion outside the core. To intuitively understand the coating thickness, a low magnification SEM image of PCU is given in Fig. 6f. The coating part with a thickness of about 15 μm and urea part were clearly exhibited, and the two parts were compactly connected. The coating thicknesses of other coated urea were similar due to the same coating rates used.

**Figure 7:**
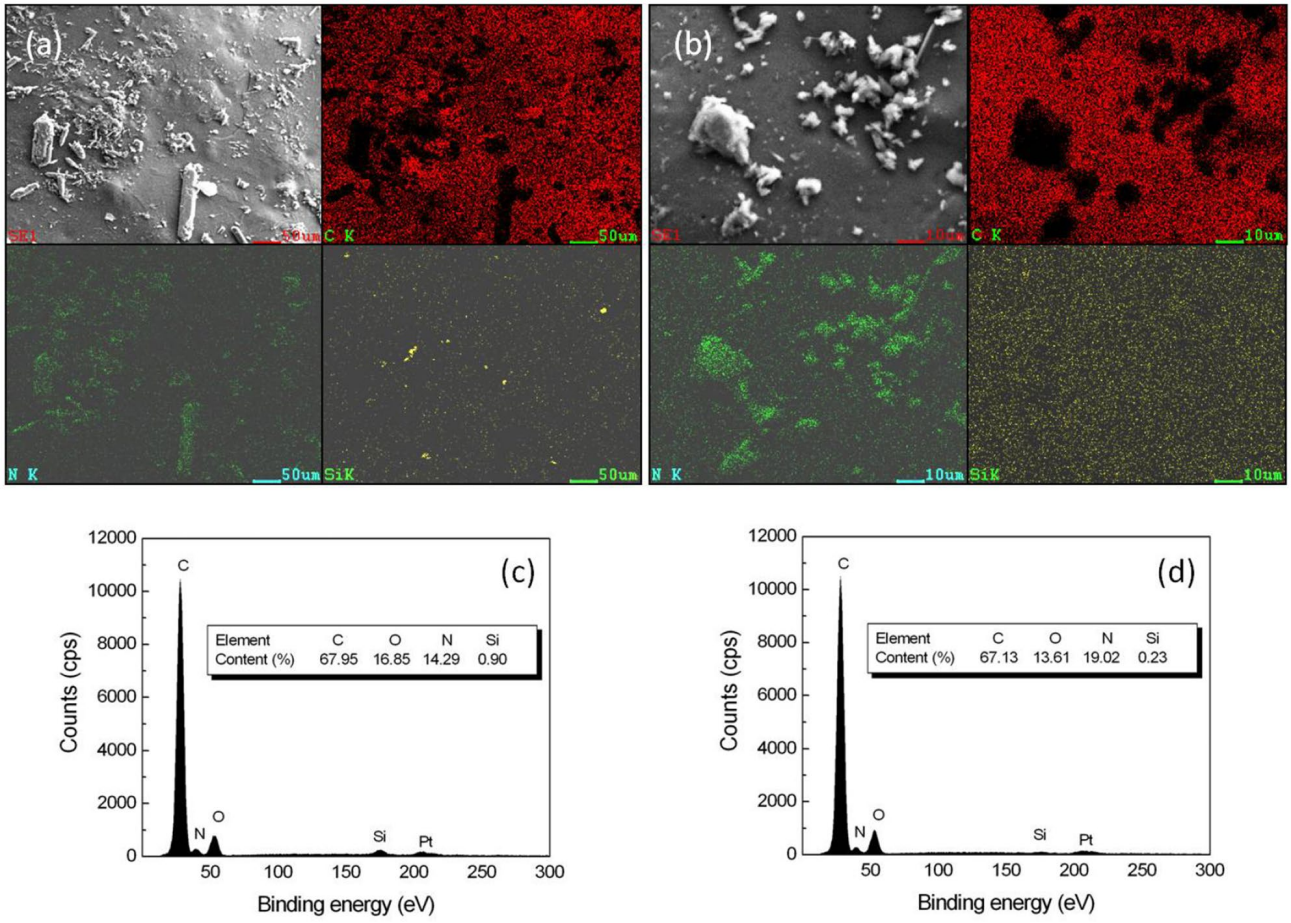
SEM images of surface morphologies and corresponding EDS mappings of C, N, Si of coatings, PU/2 wt% POSS-BEN nanocomposite PCU/BEN2 (**a**) and PU/2 wt% POSS-PEG nanocomposite (PCU/PEG2) (**b**) at × 1000 magnification, and EDS spectra of PCU/BEN2 (**c**) and PCU/PEG2 (**d**).

To understand the surface elemental distribution on coated urea, SEM images and corresponding EDS mappings of C, N and Si were shown in Fig. 7a,b. SEM results revealed a fairly rough surface, and the N element on the surface mainly came from urea left by the collision of particles during coating process. The existence of Si element indicated that POSS was successfully composited with polyurethane. For the coating with POSS-PEG, the EDS elemental maps revealed a uniform distribution of Si on the surface of polyurethane matrix, confirming that the POSS-PEG did not form detectable aggregates. The difference is that in the case of the coating with POSS-BEN, the surface did not cover well with Si but some large particles existed in the coating. The EDS spectra, shown in Fig. 7c,d, illustrated that approximately 0.23 wt% (PCU/PEG2) and 0.90 wt% (PCU/BEN2) of Si element emerged on the surface of coating, which was resulted from the different Si content of POSS itself.

### Surface hydrophobicity of coatings

**Figure 8:**
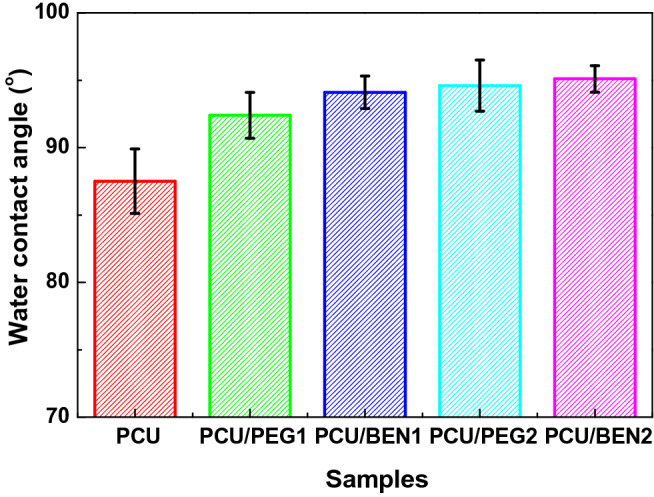
Water contact angles of coating surfaces. PU/1 wt% POSS-PEG nanocomposite (PCU/PEG1), PU/1 wt% POSS-BEN nanocomposite (PCU/BEN1), PU/2 wt% POSS-PEG nanocomposite (PCU/PEG2), PU/2 wt% POSS-BEN nanocomposite (PCU/BEN2) and pure polyurethane (PCU).

Considering POSS is a derivative of organosilicon compound for lowering free energy of polymer^40^, the prepared coatings were analyzed to see whether the surface property was changed. The static water contact angles of coatings are shown in Fig. 8. It was found that the values of PCU/BEN were a little bigger than pure PCU and PCU/PEG. For example, the water contact angles of PCU and PCU/BEN2 were 81° and 93°, respectively, exhibiting a hydrophobic feature increase after the addition of 2 wt% POSS-BEN. The presence of POSS in PU matrix provided a thermodynamic driving force for its migration from inner to surface, but the relatively higher contact angle of PCU-BEN was due to the lack of hydrogen bonding sites. This increased hydrophobicity was also related with the physical crosslinking points^41^. Therefore, the existence of POSS cage and three-dimensional network structure had an influence on the surface property of coating material. However, no significant differences were observed between nanocomposite coatings (*P* < 0.05), which was the result of the limited POSS addition. This behavior implies that the hydrophobicity of coatings was not evidently changed by increasing the POSS content in the experiment.

### Release characteristic of coated urea

**Figure 9:**
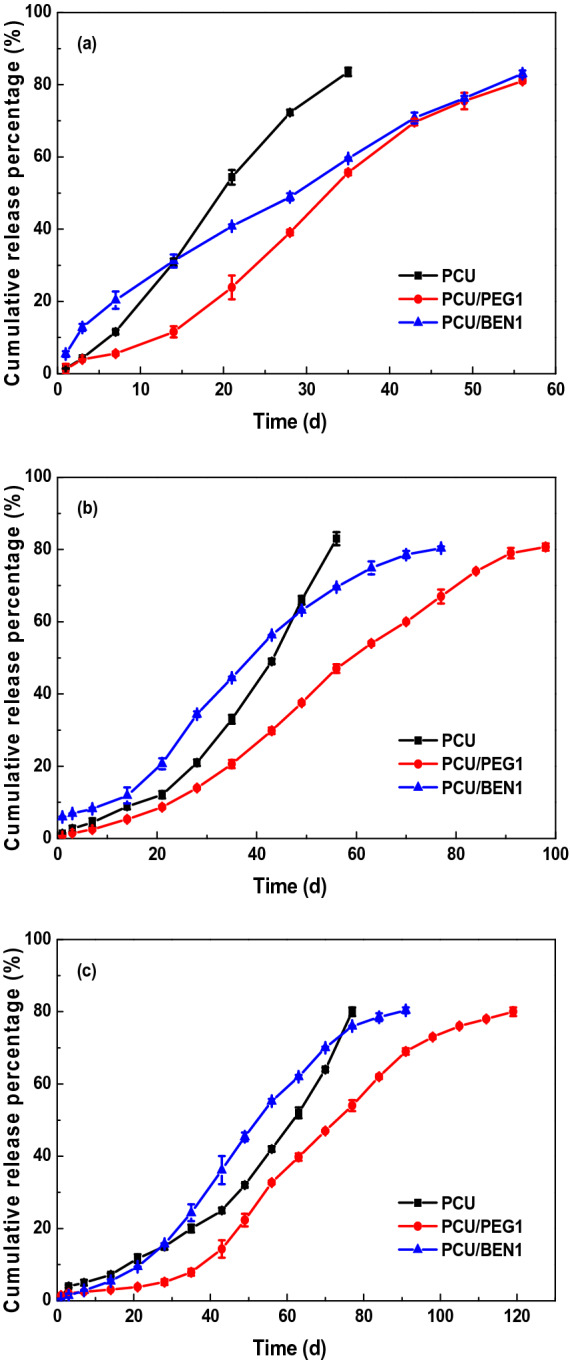
Nitrogen release behaviors of coated urea with different POSS contents at three coating rates (**a**) 2 wt%, (**b**) 2.5 wt% and (**c**) 3 wt%. PU/1 wt% POSS-PEG nanocomposite (PCU/PEG1), PU/1 wt% POSS-BEN nanocomposite (PCU/BEN1) and pure polyurethane (PCU).

The nitrogen release behaviors of coated urea in water at 25 °C were measured. The effects of POSS with different vertex groups on the nitrogen release behaviors of PCUs were studied and the results are shown in Fig. 9. As seen from Fig. 9a, the initial release rates of nitrogen were similar at 1.44%, 5.42% and 1.44%, respectively, implying that most of urea granules were covered by coating materials even when the coating rate was as low as 2 wt%. The cumulative nitrogen release curves presented different patterns, sigmoidal (S) shape for PCU/PEG1 and linear (L) shape with an initial ‘burst’ of 15% for PCU/BEN1, respectively, demonstrating that the vertex group type of POSS had a significant effect on nitrogen release characteristic. Compared with PCU, the nutrient release period of nanocomposite coatings was increased by 23 d. The thickness is another factor to tune the nitrogen release characteristic of coated fertilizer^42^. In the case of PCU/PEG1, the total nitrogen release rate with 2 wt% coating rate reached 80% near 60 d. The urea with 2.5 wt% coating rate released still less than 60% after the same time seen in Fig. 9b, and the 28-day cumulative release rate with 3 wt% coating rate was only 4.7% seen in Fig. 9c, indicating the more the coating, the lower the nutrient release rate and the longer the release period. The results for coated urea with 1 wt% POSS-BEN showed a similar trend. With the increasing of coating rate, the coating material effectively stopped the migration of water molecule and the diffusion of nutrient, and the cumulative nitrogen release curve of PCU/BEN1 began to present a sigmoidal pattern, which was another result of enhanced effect of POSS. The effect of POSS content on the nitrogen release behavior at the coating rate of 2 wt% was also investigated and shown in Fig. S3. The results of POSS/PEG1 and other nanocomposite coatings for the controlled releasing of urea are summarized in Table 2. It is found that POSS/PEG1 was a good candidate for the ultrathin coating of fertilizer. There is an exciting possibility that the release characteristic of coated urea is tunable to be in line with crop growth by varying coating rate and vertex group type of POSS.

**Table 2:** The results of PU/1 wt% POSS-PEG nanocomposite (PCU/PEG1) and other nanocomposite coatings for coated urea.

Polyurethane	Modifier	Coating rate (%)	Nitrogen release period (d)	References
Soybean oil-based	PEG-intercalated bentonite	3.7	74	^22^
Starch-based	Hydrophobic montmorillonite	3	49	^43^
Castor oil-based	Montmorillonite	4	~ 30	^44^
Pig fat-based	Superhydrophobic nano Fe_3_O_4_	3	38	^45^
Castor oil-based	POSS	2	60	This work

## Conclusions and future research directions

Castor oil-based polyurethane nanocomposite thin coatings were successfully prepared via in-situ polymerization by incorporating two types of polyhedral oligomeric silsesquioxanes, POSS-PEG and POSS-BEN, respectively, to develop controlled release fertilizer. The liquid POSS-PEG was evenly dispersed on the surface of coating due to the long and flexible PEG segment similar to castor oil, while the solid POSS-BEN was not well-dispersed as a result of benzene ring being hydrophobic and inert to polyurethane. The mechanical and thermal stability of coatings were improved as a result of the formation of physical crosslinking points after the addition of POSS. The coating PCU/PEG showed much better controlled release performance than PCU/BEN and pure PCU, and the release pattern and period were tunable by the vertex group type of POSS and coating rate of urea. In terms of practical application in agriculture, it is necessary to further study the nitrogen release in slightly acidic and alkaline environments. The POSS with different functional groups is going to optimize the polyurethane for coated fertilizer. In addition, more attention will be paid to the leaching study, so as to provide a release mechanism and give a basis for the performance optimization of nanocomposite coatings. All in all, the very thin nanocomposite coatings not only open up a new way to the property improvement of bio-based polyurethane coated fertilizers, but also extend other applications of polymer/POSS hybrid materials.

## Experimental section

### Materials

Castor oil was supplied by Tianjin Fucheng Chemical Reagent Factory, China. Polyaryl polymethylene isocyanate (PAPI, Desmodur 44V20L) was supplied by Bayer, Germany. Microcrystalline wax was supplied by Cangzhou Forest Wax, China. The regular urea granule containing 46.6% N with 2–4 mm diameter was supplied by Shandong Hualu-hengsheng Chemical Co., Ltd. China. POSS-PEG (product number PG1190, M_w_ = 5576 g/mol), a viscous liquid, was supplied by Hybrid Plastics, USA. Octaphenyl POSS (denoted by POSS-BEN), a white powder, was supplied by Shanghai Macklin Biochemical Co., Ltd. China.

### Preparation of coated urea

18 g of castor oil, 0.3 g of microcrystalline wax and 0.3 g of POSS (or 0.6 g) were mixed at 70 °C, and then the mixture and 12 g of PAPI were stirred evenly to form the coating liquid. 1 kg of urea granules in a coating machine were preheated to 70 °C. 10 g of the coating liquid was sprayed uniformly onto the urea surface and cured for about 5 min to form the coated urea with 1 wt% coating rate. After repeating the above coating process for 2 or 3 times, the coated urea granules with different coating rates were cooled to room temperature, and used for analysis. To analyze Dynamic Mechanical Analysis (DMA) of coating material, the formed coating liquid was poured into a polytetrafluoroethylene mold (20 cm × 20 cm × 0.5 cm) and kept in an oven at 70 °C overnight to conduct curing of polyurethane. Table 3 summarizes the materials and corresponding abbreviations.

**Table 3:** Materials and corresponding abbreviations.

Material	Abbreviation
Polyaryl polymethylene isocyanate	PAPI
Polyurethane	PU
Polyhedral oligomeric silsesquioxane	POSS
Poly(ethylene glycol)	PEG
POSS with eight PEG groups	POSS-PEG
POSS with octaphenyl groups	POSS-BEN
PU coated urea	PCU
PU/1 wt% POSS-PEG nanocomposite coated urea	PCU/PEG1
PU/1 wt% POSS-BEN nanocomposite coated urea	PCU/BEN1
PU/2 wt% POSS-PEG nanocomposite coated urea	PCU/PEG2
PU/2 wt% POSS-BEN nanocomposite coated urea	PCU/BEN2

### Characterization

The chemical structure of POSS was analyzed by Attenuated total reflection-Fourier transform infrared spectroscopy (ATR-FTIR)^46^. ATR-FTIR was recorded on a BRUKER TENSOR 27 spectrometer (Bruker Optics, Germany) in a scan range from 4000 to 500 cm^−1^ with a resolution of 4 cm^−1^.

The coated urea was cut up and immersed into a water bath so that urea was dissolved to obtain the coating. The coating was cleaned with deionized water and then oven-dried at 35 °C for 10 h^47^. Finally, the obtained coating was further analyzed by thermogravimetry and derivative thermogravimetry (TG/DTG).

TG/DTG analysis was performed on a NETZSCH STA449C simultaneous thermal analyzer (Selb, Germany). Samples with a weight of ~ 5 mg were heated from 40 to 600 ºC at a heating rate of 10 ºC/min under flowing argon atmosphere^47^.

DMA was carried out on a TA Q800 dynamic mechanical analyzer (New Castle, Delaware, USA) in the temperature range from − 100 to 150 ºC at the frequency of 1 Hz.

The water contact angle was determined using a HARKE-SPCAX2 goniometer (Beijing, China).

The coating was observed by using a Zeiss Supra 55 field emission scanning electron microscope (SEM, Carl Zeiss, Germany) equipped with an energy-dispersive X-ray spectroscopy (EDS) at an accelerating voltage of 20 kV. The coated urea was split into two halves, and then adhered to the sample holder with double side adhesive tape. The sample was sprayed with gold before observation^46^.

### Nitrogen release of coated urea

Nitrogen release of the prepared coated urea was evaluated based on International Standard ISO 18644. The detailed process is as follows: 10 g of the coated urea sample was immersed in 200 mL of distilled water in a plastic bottle. The temperature of incubation was 25 ºC. The solution in the plastic bottle was removed after a certain interval (1 d, 3 d, 7 d, 14 d, 21 d, 28 d…), and replaced by another 200 mL of distilled water until N release rate reached 80%. The differential release rate was measured according to the following equation ^46^.

1 $$Differntial\;release\;rate\;(\% ) = \frac{{D_{7} - D_{1} }}{6} \times 100$$

where *D*_*7*_ is the cumulative release rate in the first seven days and *D*_*1*_ is the release rate on the first day.

### Statistical analysis

Statistical analysis was conducted using Excel 2010 and SAS 9.2 (SAS Institute, Cary, NC, USA). Three duplicates were conducted for each sample. Comparison among samples was evaluated by one-way analysis of variance (ANOVA), followed by Tukey test at a significance level of *P* < 0.05. All data were expressed as mean ± standard deviation (SD).

## Supplementary Information

Supplementary Information
