# Supplementary material for: Bio-based polyurethane nanocomposite thin coatings from two comparable POSS with eight same vertex groups for controlled release urea
Source: Sci Rep. 2021 May 10;11:9917. doi: 10.1038/s41598-021-89254-9 (PMC8110765; doi:10.1038/s41598-021-89254-9)
Supplement: Supplementary file 1 — Supplementary Information [file 41598_2021_89254_MOESM1_ESM.docx]

**Supplementary Information**

Bio-based polyurethane nanocomposite thin coatings from two comparable POSS with eight same vertex groups for controlled release urea

Lixia Li^†1,2*^, Meng Wang^†3^, Xiandong Wu^1,3^, WenpingYi^1,2^ & Qiang Xiao^1,2*^

^1^Institute of Plant Nutrition and Resource, Beijing Academy of Agriculture and Forestry Sciences, Beijing 100097, China;

^2^Research Center of Beijing Municipal Slow and Controlled Release Fertilizers Engineering Technology, Beijing 100097, China;

^3^School of Nuclear Science and Technology, University of South China, Hengyang, Hunan 421001, China

*Correspondence to: Lixia Li (E-mail: [ashleyllx@163.com](mailto:ashleyllx@163.com));

Qiang Xiao (E-mail: [xqiang1978@163.com](mailto:xqiang1978@163.com))

**Characterization.** The coatings were analyzed by Attenuated total reflection-Fourier transform infrared spectroscopy (ATR-FTIR) and XRD. ATR-FTIR was recorded on a BRUKER TENSOR 27 spectrometer (Bruker Optics, Germany) in a scan range from 4000 cm^-1^ to 500 cm^-1^ with a resolution of 4 cm^-1^. XRD was recorded.

**XRD of three coatings.** Pure PCU and nanocomposites were analyzed by XRD and the results were shown in Fig. S1. The WAXD diffractograms for three coatings are very similar. Two main amorphous halos are present, denoting a microphase separated morphology at 2θ = 19.8° and 2θ = 41°. The halo around 2θ = 19.8° is typical for PU materials. No crystallite reflections appeared after the reaction. It is resulted from the good dispersion of POSS in the polyurethane coating, especially for POSS-PEG. Furthermore, considering the microstructure of nanocomposite depended on POSS content, the maximum of POSS content was 2 wt% in the manuscript and thus the change was not clear.

**Figure S1.** XRD patterns of three coatings

**FTIR of three coatings.** FTIR spectra of three coatings are shown in Fig. S2. Two bands at 2926 and 2874 cm^-1^ are due to –CH_2_ stretching vibration. The characteristic peaks of urethane in polyurethane coatings exist. The characteristic peak at 1735 cm^−1^ corresponds to −C=O stretching vibration, and the peak at 1223 cm^−1^ is related to −CO− stretching vibration. In addition, the characteristic peaks at 1540 cm^−1^ and 3330 cm^−1^ are identified as the bending vibration and stretching vibration of −NH, respectively.

**Figure S2.** FTIR spectra of three coatings.

**Release characteristic of coated urea.** Fig. S3 shows the effect of POSS content on the nitrogen release behavior at the coating rate of 2 wt%. Regardless of the vertex group on POSS, with the increase of POSS content up to 2 wt%, the initial release rate of nitrogen increased and the nitrogen release period decreased, demonstrating that at the coating thickness of around 15 µm, some POSS particles of the coatings acted as a porogen due to the weak interfacial adhesion between POSS and polyurethane. Compared with the agglomerated POSS-BEN, the well-dispersed POSS-PEG into polyurethane developed a more homogeneous coating and contributed to a relatively better controlled release property.After the consideration of cost and property, the POSS content of 1 wt% is recommended.







**Figure S3.** Nitrogen release behaviors of coated urea with different POSS contents at the coating rate of 2 wt% (a) PCU/PEG, (b) PCU/BEN.
